# Neonatal Brucellosis in an Extremely Preterm Infant: A Fatal Case Report

**DOI:** 10.1155/crdi/4095776

**Published:** 2025-11-03

**Authors:** Muhammad Takhman, Orabi Hajjeh, Moath Hattab, Reem Shihab, Hadeel Atout, Rabee Adwan, Mamoun A. T. Ibaideya

**Affiliations:** ^1^Department of Medicine, Faculty of Medicine and Health Sciences, An-Najah National University, Nablus, State of Palestine; ^2^Department of Neonatology, Palestine Medical Complex Hospital, Ramallah, State of Palestine; ^3^Department of Infectious Diseases, Makassed Hospital, East Jerusalem, State of Palestine; ^4^Department of Clinical Microbiology, Palestine Medical Complex Hospital, Ramallah, State of Palestine

**Keywords:** *Brucella melitensis*, congenital brucellosis, neonatal sepsis, preterm birth, transplacental transmission, zoonotic infection

## Abstract

**Background:**

Brucellosis is a globally significant zoonotic disease, but congenital brucellosis remains exceedingly rare. It is primarily transmitted transplacentally from an infected mother to the fetus, often presenting with nonspecific signs that mimic neonatal sepsis. The condition poses diagnostic and therapeutic challenges, particularly in endemic regions where early identification is crucial to improving neonatal outcomes.

**Case Presentation:**

We report the case of a 19-year-old gravida 1, para 0 female with a history of treated *Brucella* infection at 12 weeks of gestation. She experienced preterm premature rupture of membranes (PPROM) at 24 weeks and 2 days and delivered a female infant via spontaneous vaginal delivery. The neonate, weighing 725 g at birth, presented with cyanosis, respiratory distress, and unstable vital signs. Initial workup suggested early onset sepsis, prompting empirical antibiotic therapy. However, on Day 7, blood cultures confirmed the presence of *Brucella melitensis* and *Brucella abortus*, establishing the diagnosis of congenital brucellosis. The neonate's clinical course was complicated by intraventricular hemorrhage, necrotizing enterocolitis, multiple episodes of sepsis, and cardiac arrests. Despite intensive medical management, including antimicrobial therapy with rifampicin and gentamicin, the infant succumbed to multiple organ failure on Day 39 of life.

**Conclusion:**

This case highlights the diagnostic challenges of congenital brucellosis and underscores the importance of considering *Brucella* infection in neonates presenting with unexplained sepsis, especially in endemic regions. Early recognition and targeted therapy are essential to improve neonatal outcomes. Enhanced screening protocols for pregnant women in high-risk areas, coupled with heightened clinical suspicion in neonates with maternal brucellosis exposure, could facilitate timely diagnosis and treatment.

## 1. Case Presentation

**Figure 1:**
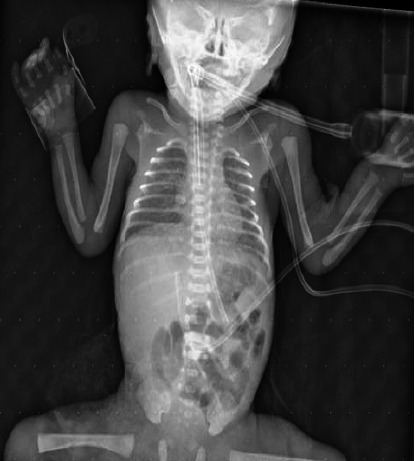
The initial chest x-ray demonstrated diffuse reticulogranular opacities with air bronchograms, consistent with neonatal respiratory distress syndrome (RDS). There was no evidence of pneumothorax or congenital lung anomalies.

A 19-year-old asymptomatic gravida 1, para 0, female experienced preterm premature rupture of the membranes (PPROM) at 24 weeks and two days of gestation and was admitted to hospital. The pregnancy was uneventful except for documented Brucella infection at 12 weeks of gestation and prescribed a 6-week course of antibiotics consisting of a combination of doxycycline and rifampin. However, the patient was noncompliant and discontinued the medications after a short period, without completing the full course or attending follow-up. The pregnancy was uneventful except for documented Brucella infection at 12 weeks' gestation treated with a 6-week course of antibiotics with no complaint. She delivered a female infant by spontaneous vaginal delivery. The infant had cyanosis on initial presentation along with irregular breathing, Apgar scores at birth were 6 and 8, after 1 and 5 min, respectively. So, the infant had to be intubated and transferred to the neonatal intensive care unit (NICU) for further management (Figure1). On initial examination, the patient had weight, 725 g; head circumference, 26 cm; length, 38 cm; and unstable vital signs. The patient was connected to mechanical ventilation on synchronized intermittent mandatory ventilation (SIMV) mode, first dose of surfactant was given, and she was on paracetamol and caffeine for apnea prophylaxis. Blood specimens for cultures were obtained, then the neonate was treated empirically with ampicillin (18 mg intravenous [IV] every 12 h) and gentamicin (3.5 mg IV every 48 h) as a result of early onset of sepsis suspicion. Blood tests were significant for high C-reactive protein. Complete blood count (CBC) and random blood sugar were normal. On Day 5, the patient's white blood cell (WBC) count was elevated to 42 × 10^9^/L, prompting an upgrade in antibiotics to piperacillin/tazobactam (35 mg IV every 12 h) and amikacin (5 mg IV every 24 h), to provide broader Gram-negative and anaerobic coverage.

Echocardiography was done for the patient and was free apart from small patent foramen ovale. On Day 7, blood cultures revealed the presence of Brucella. The father confirmed a maternal history of treated Brucella infection before 3 months of delivery. The mother was advised to avoid breastfeeding the baby. Targeted therapy for brucellosis was initiated by adding rifampicin (14.5 mg orally every 24 h) and gentamicin (3 mg IV every 36 h) to the existing antibiotic regimen, while amikacin was discontinued to minimize aminoglycoside toxicity and avoid redundancy in coverage. It is important to note that the first blood culture, taken from the baby on Day 1, was conducted before breastfeeding began and prior to the administration of any antibiotics. Test results revealed the following: blood culture showed growth of *Brucella melitensis* (titer 1/320) and *Brucella abortus* (titer 1/320). The maternal Brucella IgM titer was 41 (normal value: < 11), while the baby's IgM titer was 3. The low Brucella IgM titer in the infant is consistent with the known limitations of serological testing in neonates, particularly in extremely preterm infants who may have an underdeveloped immune response. Despite the low IgM, blood cultures confirmed active infection, which remains the gold standard for diagnosis [1].

**Figure 2:**
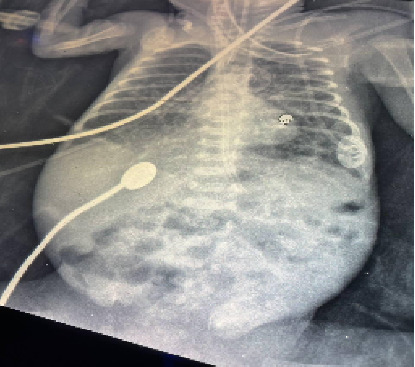
The x-ray shows pneumatosis intestinalis with multiple small air bubbles along the bowel wall, suggestive of necrotizing enterocolitis.

On Day 11, the patient had intraventricular hemorrhage (IVH) grade III detected on transfontanelle ultrasound (TFU) and persisted throughout his course. The day after, feeding was withheld due to vomiting and abdominal distension that was later revealed to be necrotizing enterocolitis (Figure 2). During the patient course, she had seven blood transfusions for anemia, and she also had many changes in her antimicrobial drug following elevation in inflammatory markers multiple time for sepsis of bacterial and fungal sources. On Day 38, the patient had cardiac arrest but was resuscitated and started in dopamine infusion, perforated necrotizing enterocolitis was detected on x-ray, and intra-abdominal drain was applied. On the next day, she had another arrest but she was resuscitated after 2 cycles and calcium gluconate infusion. Tragically, she suffered another cardiac arrest, and despite administering three dopamine boluses, resuscitation efforts were unsuccessful. The patient was pronounced dead.

## 2. Introduction

Brucellosis, a common worldwide zoonotic infection, transmitted form animals to humans through contact with infected animals or contaminated animal product. Congenital brucellosis on the other hand is extremely rare and occurs through transplacental transmission from the mother to the fetus. Congenital brucellosis represents a diagnostic challenge especially in the endemic areas, as the clinical manifestations are usually nonspecific mirroring a sepsis-like picture pointing out the importance of detailed history of the maternal history of ingesting unpasteurized milk or contact with animals.

Here, we present the case of a 24-week-old infant with respiratory distress and a sepsis-like presentation, ultimately diagnosed with congenital brucellosis through blood culture.

## 3. Discussion

**Figure 3:**
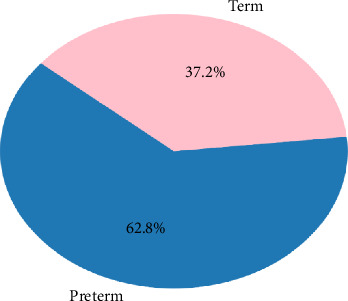
Distribution of gestational age in congenital brucellosis (*n* = 43).

Congenital brucellosis is an invisible threat, especially in brucellosis-endemic areas where Brucella species are transferred from infected mothers to unborn children through transplacental transmission with deadly consequences including spontaneous abortions, intrauterine fetal deaths, and prematurity (Figure 3) [2].

**Figure 4:**
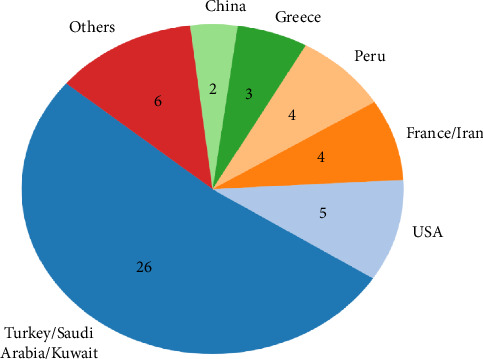
Geographic distribution of congenital brucellosis (*n* = 50).

Although pregnant women in endemic regions (Figure 4) are at risk of Brucella exposure, congenital brucellosis remains an exceptionally rare condition, with only 44 cases reported globally up to 2017 [2]. A search of subsequent publications revealed 7 additional cases reported since then, making this the 51st case (Table 1). The transmission mechanisms, clinical manifestations, and long-term outcomes associated with congenital brucellosis remain underexplored in the current literature.

Congenital brucellosis is thought to occur through transplacental transmission from a bacteremic mother or through exposure to maternal blood, urine, or genital secretions during delivery. Half of the reported cases were bacteremic at birth, supporting transplacental transmission as a likely mechanism [2]. While Brucella transmission via breast milk and blood transfusion has been documented in the literature [10, 11], our patient's positive blood culture, with the absence of breastfeeding or transfusion, points strongly to transplacental transmission as the probable route. While the clinical course and eventual outcome in our case were likely driven by the complications of extreme prematurity, the potential role of maternal Brucella infection in precipitating PPROM and subsequent preterm delivery cannot be overlooked. Several studies have identified an association between maternal brucellosis and adverse obstetric outcomes, including spontaneous abortion, intrauterine fetal demise, and preterm birth [2, 12, 13]. In this case, the incomplete treatment and absence of follow-up may have contributed to persistent bacteremia, thereby increasing the risk of vertical transmission and possibly triggering premature labor. However, due to the lack of placental histopathology or maternal serologic follow-up, a definitive causal link cannot be established.

The clinical presentation of congenital brucellosis is nonspecific, frequently manifesting as premature rupture of membranes, respiratory distress, or a sepsis-like picture with hepatosplenomegaly or jaundice as a common exam finding. Less common presentations, including myocarditis and hydrocephalus, have also been documented. These varied manifestations underscore the importance of a high index of suspicion among clinicians, especially in endemic regions or when there is a maternal history of ingesting unpasteurized milk or contact with animals. Recognizing these risk factors early can aid in early diagnosis and management [12, 13]. Noncompliance with the prescribed antibiotic regimen during pregnancy, as seen in our case, may contribute to persistent maternal bacteremia and increase the risk of vertical transmission. This highlights the importance of thorough counseling, close monitoring, and follow-up in pregnant women diagnosed with brucellosis, especially in endemic areas.

**Figure 5:**
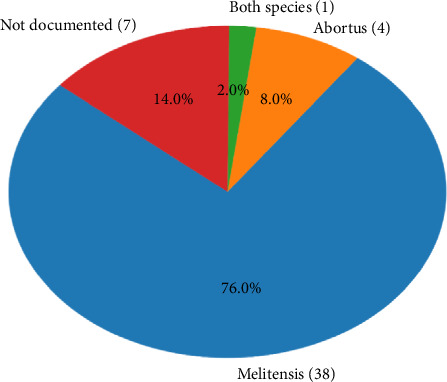
Distribution of Brucella species in congenital cases (*n* = 50).

Diagnosis of neonatal brucellosis depends primarily on positive blood cultures that isolate the Brucella organism, most commonly *Brucella meltinesis* (Figure 5), although serological tests showing elevated or rising antibody titers in either the mother or neonate and history of exposure to livestock or ingesting raw milk can also provide important diagnostic clues. In rare cases, Brucella has been recovered from blood cultures of children who have negative or insignificant antibody titers (< 1:160), making bacteremia a more reliable indicator of infection. Serological tests (serum agglutination test) are helpful in diagnosing and monitoring the disease, and titers of 1:160 or higher are significant. However, the value of these tests is low because bacteremia provides unequivocal evidence of infection. Nevertheless, the diagnosis by microbiology is delayed as Brucella grows slowly. In this context, prompt identification and early antibiotic treatment will lower the risk of serious complication, which highlights the need of knowing clinical features of neonatal brucellosis for appropriate management [1, 12, 14]. Other rapid diagnostic techniques, such as polymerase chain reaction (PCR), are emerging as valuable tools, offering improved sensitivity and faster results [15].

A treatment approach of intravenous aminoglycosides for 5–14 days with oral rifampicin and trimethoprim/sulfamethoxazole (TMP/SMX) for 6 weeks is commonly reported in literature review as a treatment for congenital brucellosis [12]. Not to forget the relative contraindication of TMP/SMX in children less than 2 months of age as it displaces indirect bilirubin from albumin, increasing the risk of jaundice and subsequent kernicterus highlighted in multiple studies.

New update in treatment for pediatric brucellosis depends on the age: where oral doxycycline and rifampicin are used for children above the age of 8. On the other hand, for those under 8 years old, a combination of oral trimethoprim, sulfamethoxazole, and rifampicin of a 6–8-week duration is advised [4]. The varying success rates of different brucellosis treatment approaches highlight the need for further research into optimal therapies for neonates. Additionally, the high recurrence rates, which can be partially attributed to incomplete eradication of the infection or reinfection through breast milk, along with the risk of complications, emphasize the importance of long-term follow-up for affected infants.

From a public health perspective, eradicating congenital brucellosis should be done through prevention and treatment. Antenatal screening in high-risk populations could facilitate early identification and management of maternal brucellosis, reducing the risk of congenital transmission. The information and awareness campaigns in the countries that suffer brucellosis should inform people on the dangers of eating unprocessed animal milk and contact with animals, as the majority of infected pregnant women reported a history of unpasteurized milk consumption or contact with animals [12]. Furthermore, improving the diagnostic methods in low-resourced areas with the use of PCR, e.g., or other rapid diagnostic measures may lead to earlier diagnosis and treatment, which would improve the health of the neonate.

In conclusion, congenital brucellosis is an extremely rare condition posing serious challenges in diagnosis, treatment, and follow-up to ensure complete eradication of the infection. Crucial efforts to implement preventative strategies and raise awareness among people and healthcare practitioners are required to minimize the disease's burden. Finally, a multidisciplinary approach that combines public health activities with clinical care will be critical to improve outcomes for afflicted newborns.

## Figures and Tables

**Table 1:** Reported cases of congenital brucellosis after 2017.

Citation	Country	Gestational age	Birth weight	Infant symptoms	Species	Therapy	Outcomes
[3]	China	Term	—	Fever for 8 days	*Brucella melitensis*	Rifampicin and sulfamethoxazole/trimethiprim (SMZ/TMP)	Chronic brucellosis with full recovery
[4]	Saudi Arabia	29 weeks	1.085 g	Severe respiratory depression, pyrexia, hypotension	*Brucella abortus* and *B. meltinesis*	Rifampin/gentamicin/ciprofloxacin. Gentamicin was changed to meropenem	Survived
[5]	Kuwait	Term	2.3 kg	Respiratory distress, meconium, aspiration syndrome, intrauterine growth retardation.	Not specified	Rifampicin and ciprofloxacin for 42 days	Survived
[6]	Saudi Arabia	30 weeks	1200 g	Severe respiratory distress	*Brucella abortus*	Rifampicin, gentamicin, and ciprofloxacin	Subsequent *Klebsiella pneumoniae* coinfection, arrested at Day 33.
[7]	China	34–36 weeks	2.0 kg	Pyrexia, shortness of breath, hepatosplenomegaly, and thrombocytopenia	*Brucella meltinesis*	Trimethoprim/sulfamethoxazole, rifampicin, ceftriaxone	Survived
[8]	Turkey	38 weeks	2700 g	Jaundice and poor sucking	*Brucella melitensis*	Gentamicin, rifampicin, and cefotaxime	Survived
[9]	Saudi Arabia	29 + 4 weeks	1.495 kg	Severe respiratory distress, hypoactive, jaundice, hypotension	Not specified	Ampicillin/gentamicin, that was changed to trimethoprim/sulfamethoxazole plus rifampin	Survived

## Nomenclature

PPROM: Preterm premature rupture of membranes
NICU: Neonatal intensive care unit
SIMV: Synchronized intermittent mandatory ventilation
CBC: Complete blood count
WBC: White blood cell
IV: Intravenous
TFU: Transfontanelle ultrasound
IVH: Intraventricular hemorrhage
PCR: Polymerase chain reaction
TMP/SMX: Trimethoprim/sulfamethoxazole
